# *Pogostick*: A New Versatile *piggyBac* Vector for Inducible Gene Over-Expression and Down-Regulation in Emerging Model Systems

**DOI:** 10.1371/journal.pone.0018659

**Published:** 2011-04-14

**Authors:** Bin Chen, Steven Hrycaj, Johannes B. Schinko, Ondrej Podlaha, Ernst A. Wimmer, Aleksandar Popadić, Antónia Monteiro

**Affiliations:** 1 Department of Ecology and Evolutionary Biology, Yale University, New Haven, Connecticut, United States of America; 2 Institute of Entomology and Molecular Biology, Chongqing Normal University, Chongqing, People's Republic of China; 3 Department of Biological Sciences, Wayne State University, Detroit, Michigan, United States of America; 4 Department of Developmental Biology, Georg-August-University Göttingen, Göttingen, Germany; Michigan State University, United States of America

## Abstract

**Background:**

Non-traditional model systems need new tools that will enable them to enter the field of functional genetics. These tools should enable the exploration of gene function, via knock-downs of endogenous genes, as well as over-expression and ectopic expression of transgenes.

**Methodology:**

We constructed a new vector called *Pogostick* that can be used to over-express or down-regulate genes in organisms amenable to germ line transformation by the *piggyBac* transposable element. Pogostick can be found at www.addgene.org, a non-profit plasmid repository. The vector currently uses the heat-shock promoter *Hsp70* from *Drosophila* to drive transgene expression and, as such, will have immediate applicability to organisms that can correctly interpret this promotor sequence. We detail how to clone candidate genes into this vector and test its functionality in *Drosophila* by targeting a gene coding for the fluorescent protein DsRed. By cloning a single *DsRed* copy into the vector, and generating transgenic lines, we show that *DsRed* mRNA and protein levels are elevated following heat-shock. When cloning a second copy of *DsRed* in reverse orientation into a flanking site, and transforming flies constitutively expressing *DsRed* in the eyes, we show that endogenous mRNA and protein levels drop following heat-shock. We then test the over-expression vector, containing the complete cDNA of *Ultrabithorax* (*Ubx*) gene, in an emerging model system, *Bicyclus anynana*. We produce a transgenic line and show that levels of *Ubx* mRNA expression rise significantly following a heat-shock. Finally, we show how to obtain genomic sequence adjacent to the *Pogostick* insertion site and to estimate transgene copy number in genomes of transformed individuals.

**Significance:**

This new vector will allow emerging model systems to enter the field of functional genetics with few hurdles.

## Introduction

With the completion of a number of genome projects, probing the function of individual genes has become a main challenge. Non-traditional model systems, in particular, need new tools that will enable them to enter the field of functional genetics. These tools should enable the exploration of gene function, via knock-downs of endogenous genes, as well as over-expression and ectopic expression of transgenes.

The most popular development in the field has been the use of dsRNA to knock-down homologous genes via RNA interference (reviewed in [1]). RNAi was first discovered in plants as a mechanism for post-transcriptional gene silencing [2] and is now known to exist also in fungi and animals [3], [4], [5]. RNAi depends on the successful delivery of dsRNA into the cytoplasm of target cells and current delivery methods include viral transformation, lipofection, electroporation, direct injection, biolistics, soaking, and feeding [6], [7]. Once inside a cell, the ability of RNAi to spread to other cells to produce systemic effects varies across species and tissues and it is still unclear which spreading mechanisms are used outside of *C. elegans* [8], [9], [10]. So, while successful systemic RNAi knock-downs have been achieved in hemipterans [11] and various beetles [10], [12], [13], systemic spreading does not readily happen in *Drosophila*, *Anopheles*, or *Aedes* [10], and appears to work in limited tissues in the Lepidoptera [14], [15], [16], [17]. A way to overcome the limitation of systemic spreading is to induce the RNAi mechanism directly inside the cells, using transgenesis [18].

In addition to RNAi-mediated knock-downs, ectopic expression and over-expression of genes are also informative regarding gene function. Ectopic expression can be useful to test gene sufficiency in the development of a trait, and over-expression can test whether reverse phenotypes, relative to those observed from the knock-down experiments, are produced. For these experiments, transgenesis is usually required. Heritable over-expression and knock-downs mediated by transgenesis have been successfully applied for determining gene function in various organisms, e.g. the former in tobacco [19], mosquito [20] and mouse [21], and the latter in nematode [22], *Drosophila* [23], and mouse [24].

The development of new transgenic systems for emerging model systems depends, to a large extent, on the availability of transformation vectors of wide applicability across taxa. It also depends on the versatility of these vectors, i.e., being designed for use in a variety of gene function assays. The transposable element *piggyBac* is arguably one of the most promiscuous transposable elements discovered to date. Transposons related to *piggyBac* have been found in the genomes of almost all eukaryotes [25], and *piggyBac* derived vectors have now been used to transform a range of vertebrate and invertebrate species [26], [27], [28], [29], [30], [31], [32]. Given these qualities we chose it as the vehicle around which we designed a new versatile vector, *Pogostick,* which can be used both to over-express transgenes, as well as down-regulate endogenous genes, in a controlled temporal fashion, by means of a heat-shock. We note that *piggyBac* vectors that can either over-express or down-regulate genes in *Bombyx* have been previously described [33], [34], but these vectors were not specifically designed to serve both functions, nor were they designed to function with multiple genes, and described in sufficient detail to be of general use to the emerging model system community.

After a brief description on how *Pogostick* was constructed, we detail its main features and how candidate genes can be inserted into it for use in either over-expression or down-regulation experiments. We subsequently test *Pogostick* in functional genetic experiments with *Drosophila.* In particular, we document 1) the over-expression of the fluorescent protein DsRed at the larval and pupal stages of development, after a heat-shock is administered, and 2) the down-regulation of DsRed in a line constitutively expressing this gene in the eyes, also following a heat-shock. We document *DsRed* regulation both by directly monitoring *DsRed* mRNA levels using q-PCR and by monitoring florescent levels of the protein using fluorescent microscopy. We then test *Pogostick* in an emerging model system, the butterfly *Bicyclus anynana*. We construct a vector containing the previously cloned complete coding sequence of *Ultrabithorax (Ubx)* from another butterfly, *Junonia coenia,* produce a new transgenic line, and describe the transcriptional profile of *Ubx* mRNA following a single heat-shock in *B. anynana* larvae. Finally, we show how to obtain sequences flanking *Pogostick* genomic insertions, how to determine insertion number in transformed individuals, and how to set-up homozygous lines.

## Materials and Methods

### Pogostick vector construction

**Figure 1:**
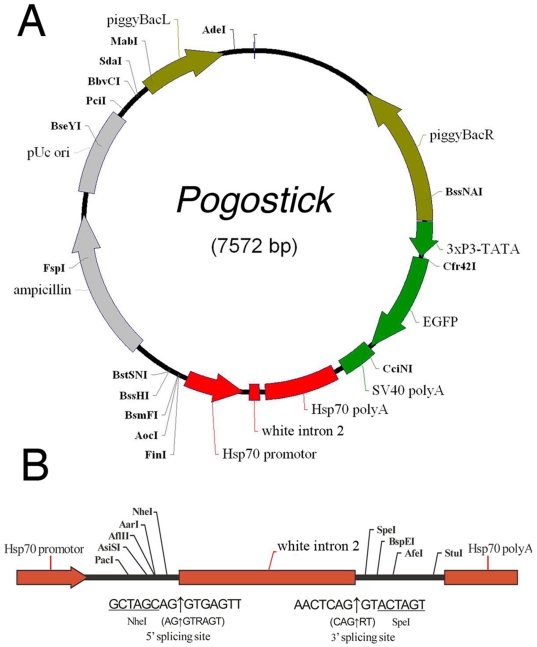
Schematic representation of the *Pogostick* vector. A) Complete vector with restriction sites; B) Detail of the two multiple cloning sites in *Pogostick.* The sequences at the junction of the *white* intron 2 of *Drosophila* are shown below with the arrows indicating the 5′ and 3′ splicing sites. The consensus sequences for the 5′ and 3′ splicing sites are shown in parenthesis; R = A or G.

The new versatile *piggyBac* vector, *Pogostick* (7572 bp; Fig. 1), was constructed with three steps. Firstly, the *EGFP* fragment (700 bp) in the plasmid *pBac[3xP3-DsRed, HS-EGFP*] [35] was replaced with the 74 bp *White* intron 2 (X02974) of *Drosophila melanogaster* and flanking MCSs (*PacI*, *AsiSI*, *AflII*, *AarI* and *NheI* in 5′end; *SpeI*, *BspEI*, *AfeI* and *StuI* in 3′ end) as a spacer/linker, cut and inserted with the flanking *HpaI* and *NotI* (Fig. 1B). Then, the 600 bp fragment containing *D. melanogaster Hsp70* promoter/*white* intron 2/*Hsp70* polyA, from the newly-built *pBac[3xP3-DsRed, HS-white intron 2]*, was cloned into the transposon *piggyBac*-based vector *pBac[3×P3-EGFPafm]* [36], cut and inserted both with the unique *AscI*. The *Hsp70* promoter directs transcription from piggyBacL to SV40 polyA in the new *pBac[3×P3-EGFPafm, HS-white intron 2]* (Fig. 1A). Finally, the 2455 bp containing the plasmid origin of replication (pUC ori) and ampicillin resistant gene (PAP) in *the pBac[3×P3-EGFPafm, HS-white intron 2]* was moved from between piggyBacR and piggyBacL to between piggyBacL and the *Hsp70* promoter. Unique restriction enzyme (RE) sites, *PciI*, *BbvCI*, and *SdaI,* were inserted between piggyBacL and pUC ori and a different set of unique sites, *FinI*, *AocI*, *BsmFI*, *BssHI* and *BstSNI*, were inserted between the Hsp70 promotor and the ampicillin resistant gene (Fig. 1A).

### Using Pogostick to make new vectors for candidate gene over-expression and down-regulation: an example with DsRed and Ubx

#### *DsRed* cDNA

The 681 bp of *DsRed* complete cDNA (AY569780.1) was amplified with PCR from *pBac[3×P3−DsRedaf]* [36] using forward primer AGGCCTCTAGAATGGTGCGCTCCTCCAAGAACGTCAT and reverse primer GTCCATCTAGACTACAGGAACAGGTGGTGGCGGCCCT, where the underlined sequences contain the *XbaI* recognition sequence (TCTAGA) and an additional randomly picked first five bases that work as a landing site for *XbaI*. PCR fragments were first digested with XbaI and purified with QIAprep Miniprep Kit (Qiagen), before being inserted into *Pogostick* at different positions.

#### Over-expression *DsRed v*ectors

**Figure 2:**
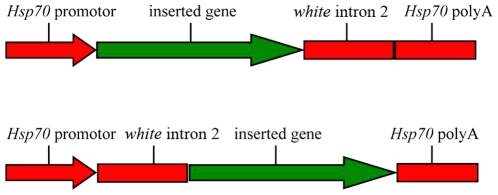
Alternative locations where a candidate gene can be inserted for over-expression experiments. Top: the gene is inserted just before the *white* intron. Bottom: the target gene is inserted just after the intron. Insertions at both positions are also possible.

Two *DsRed* over-expression vectors were constructed by cloning *DsRed* cDNA into each of the two MCS of *Pogostick*: One between the *Hsp70* promotor and the *white* intron and the other between the *white* intron and *Hsp70* polyA tail (Fig. 2). For the first vector, *Pogostick-up-1,* previously digested *DsRed* cDNA was cloned into the *NheI* restriction site in *Pogostick*. A phosphatase (Apex™ Heat-Labile Alkaline Phosphatase, Epicentre) was used for dephosphorylation of the cut vector ends prior to cloning to prevent recircularization. Competent cells (JM109, Promega) were transformed with the plasmid and grown on ampicillin selective medium. Clones were picked and confirmed to contain the insert via PCR amplification and sequencing with primers HSP1-F (TCAACTGCAACTACTGAAATCTGCCA) and HSP1-R (ACACAGATCAGCCGACTGCGAA; intron-anchored). The length for this amplicon was 833 bp. For the second vector, *Pogostick-up-2, DsRed* was cloned into *SpeI* of *Pogostick* and PCR amplification and sequencing (of picked clones) was done with primers HSP2-F (TCGCAGTCGGCTGATCTGTGTG; intron-anchored) and HSP2-R (TCGACGGATCCCCGACACCA). The length for this amplicon was 845 bp. Both plasmids, *Pogostick-up-1* and *Pogostick-up-2* were 8,259 bp long.

#### Over-expression *Ubx v*ector

We obtained a plasmid with the complete coding sequence (762 bp) of *Junonia coenia* cDNA (gi|18535619) from Sean Carroll's lab. Using primer sets Ubx-Junonia-FW: AGGCC**TCTAGA**ATGAACTCCTATTTCGAGCA and Ubx-Junonia-RV: GTCCA**TCTAGA**TTAGTGCTCGGGGTGGCCCT we PCR-amplified the complete *Ubx* sequence while adding *NheI* restriction sites (bold) at both ends of the amplicon. Then we followed the cloning steps detailed above in order to insert Ubx into the MCS of *Pogostick* immediately following the *Hsp70* promoter. We used primers Clone-RV: AACGGCATACTGCTCTCGTT and Ubx-Junonia-RV to pick up positive clones for sequence conformation.

#### Down-regulation *DsRed* vectors

**Figure 3:**
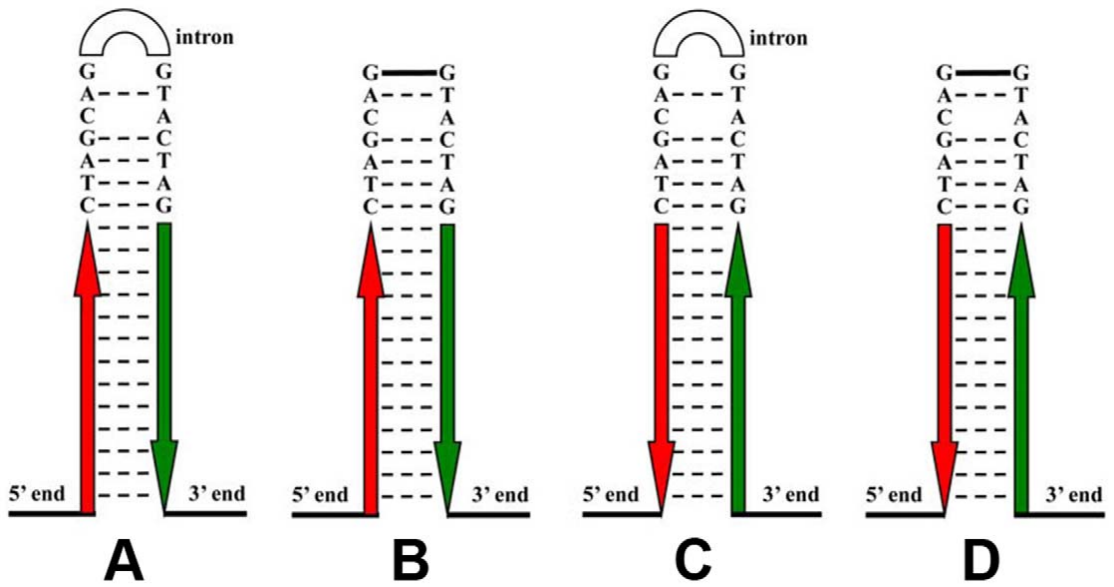
Secondary structures of alternative hairpin-loops in RNAi constructs. A and C correspond to structures before the intron is spliced and B and D correspond to structures after splicing, both when the transgene fragment is inserted into *NheI* and *SpeI* restriction enzyme sites. Red and green denote complementary sequences of the fragment with the arrows indicating 5′ to 3′ sequence orientation.

Two down-regulation vectors were constructed based on *Pogostick-up-1* and *Pogostick-up-2*. For the first vector, using *Pogostick-up-1, DsRed* cDNA was cloned into *SpeI* in reverse direction to produce *Pogostick-down-FR*, whose correct ligation was confirmed by the size (845 bp) and the sequence of the amplicon using primers HSP2-F and HSP2-R (Fig. 3A). Similarly, using *Pogostick-up-2* as the starting plasmid, *DsRed* was cloned into *NheI* in reverse direction to produce *Pogostick-down-RF*, whose correct ligation was confirmed by the size (833 bp) and sequence of the PCR amplicon using primers HSP1-F and HSP1-R (Fig. 3C). Both copies of *DsRed* cDNA were in a reverse orientation inside each vector, but each vector had a different general orientation of the sequences (compare Figs. 3A and 3C).

### Drosophila transformation and bioassays

#### *Drosophila* transformation

We tested three over-expression constructs, *Pogostick* (control vector), *Pogostick-up-1* and *Pogostick-up-2*, and two down-regulation constructs, *Pogostick*-down-FR and *Pogostick*-down-RF, by injecting each separately into wild-type *white-D. melanogaster* together with the *piggyBac* helper plasmid, *phsp-pBac* [37]. Stable lines were produced by using balancer chromosomes. *Pogostick-down-FR* and *Pogostick-down-RF* lines were then separately crossed with a *pBac[3×P3−DsRedaf]* line that constitutively expresses DsRed in the eyes and central nervous system [38], to produce transheterozygous situations. Germ line transformation and rearing of *D. melanogaster* were conducted with the methods described in [39].

#### Over-expression bioassays

About 50 2^nd^ instar larvae were separately collected from *Pogostick* (control), *Pogostick*-*up-1* and *Pogostick-up-2* lines and placed in a Petri dish on some food. Five larvae from each line were randomly picked, photographed under a fluorescent microscope (Nikon SMZ1500) and fixed in RNA*later* (Ambion). The other larvae were subject to 1.5 hours of heat-shock at 39°C in an incubator and then returned back to room temperature (∼20°C). Groups of five larvae were randomly picked at 2 hr, 6 hr, 12 hr, 24 hr, 36 hr and 48 hrs after the heat shock, respectively, photographed and then fixed in RNA*later*. The same treatment was applied to young pupae, but the heat shock period was 3 hr.

#### Down-regulation bioassays

Larvae and pupae from three lines: Homozygous *pBac[3×P3−DsRedaf]* (control), transheterozygous *Pogostick−down−FR* × *pBac[3×P3−DsRedaf]* and *Pogostick−down−RF* × *pBac[3×P3−DsRedaf]* were collected and treated as described above for the over-expression lines.

### Bicyclus transformation and bioassays

#### Bicyclus transformation

A total of 3035 eggs were injected with 25 ul of *pogostick-Ubx-up1* (1.2 ug/ul) mixed in with 25 ul of the helper plasmid (800 ng/ul). We obtained 431 hatchings (14% hatching rate). From these larvae, 145 (34%) survived to the adult stage. We crossed males and females in two separate mating cages. One cage (#1) had 43 females crossed to 30 males. The other cage (#2) had 43 females crossed with 29 males. While screening the offspring of these two mating cages we picked a single male from cage #2′with extremely bright eyes (when viewed under a fluorescent scope with a EGFP filter) and mated him with 12 virgin females. This male and two of his offspring, all with bright green eyes, were later confirmed as positive for *EGFP*. Roughly half the F2 offspring of this male (127) displayed bright stemmata (larval eyes) whereas the other half looked wild type, suggesting that the insertion was at a single locus. Several (around 30) bright-eyed heterozygous F2 individuals were crossed with each other to produce a F3 generation. All F3 individuals were photographed under the fluorescent scope. They segregated according to Mendelian ratios. From 77 females, 16 had a wild-type phenotype (expected frequency = 19), and the other females had very bright or intermediately bright eyes in approximately the predicted ratios for homozygous and heterozygous classes. Eight females and six males with the brightest eyes were selected for parents of a F4 generation in order to produce an homozygous line. We subsequently performed the qPCR experiments using offspring from this line.

#### Over-expression bioassays

Fifth instar Ubx transgenic larva were put into plastic cups and heat-shocked for 3 hrs at 39°C. Immediately after the heat-shock, 3 larvae were collected (0 hrs) whereas the rest were moved (still inside cups) to the normal rearing temp of 27°C. Three additional larvae were collected at 5, 10, 15, and 20 hours after the end of the heatshock. Three control non-heat-shocked larvae were also collected. Immediately after collection larva were decapitated and gutted. About 25 mg of tissue was then cut off and set aside for RNA extraction. These samples were kept frozen at *−*80°C until RNA extraction.

### Real-time q-PCR

Total RNA isolation from larvae and pupae kept in RNA*later* (*Drosophila*) or kept at *−*80°C (*Bicyclus*) was performed using an RNeasy Mini kit (Qiagen). RNA was treated with RNase-free DNase I (Qiagen) to eliminate genomic DNA. cDNA was reverse-transcribed from total RNA using random nanomers and using a High-Capacity cDNA Reverse Transcription Kit (Applied Biosystems). Real-time q-PCR was performed with TaqMan Universal PCR Master Mix and Custom TaqMan Gene Expression Assays in STANDARD mode using the Applied Biosystems 7500 Fast Real-Time PCR System. Eukaryotic 18 S rRNA was used as the endogenous control. Relative quantification in 2^-ΔΔC^_T_ [40] was normalized to the 18 S rRNA to indicate levels of *DsRed* and *Ubx* transcripts.

### Obtaining flanking sequence to Pogostick genomic insertions

A plasmid-rescue technique was used to obtain flanking genomic sequences for each side of the *Pogostick* insertion in the *Drosophila* lines, via two separate experiments, using genomic DNA of the transformed individuals. Briefly, each experiment used a unique restriction enzyme that cuts at one of the known ends of the ampicillin resistant and pUc ori sequence block and at multiple unknown locations throughout the genome. Genomic fragments are then circularized into plasmids by ligation, inserted into competent bacterial cells and cells grown in ampicillin selective medium. Only cells containing a plasmid with the ampicillin resistant gene and origin of replication should survive. These plasmids also contain either right or left *Pogostick* flanking genomic sequences.

Genomic DNA of transformed *Drosophila* was extracted with a Qiagen DNeasy kit and digested with *BstSNI* in order to target genomic sequences adjacent to the terminal inverted repeat (TIR) of piggyBacL. Other alternative unique vector RE sites are *BssHI*, *BsmFI*, *AocI* or *FinI*. After a plasmid mini-prep, genomic sequences were obtained using the piggyBacL-anchored sequencing primer 5′-AACAAGCTCGTCATCGCTTT-3′. Genomic sequences adjacent to piggyBacR were obtained using RE *BbvCI* (alternatively, *PciI* or *SdaI* can also be used) and the piggyBacR-anchored sequencing primer 5′-CATGAATGACGGGGAGATTT-3′. No restriction enzyme that cuts inside the transgenes should be used. The diversity of sequences obtained from multiple plasmids gives a lower estimate of *Pogostick* insertion copy number. A Southern blot (see [35]), on the other hand, will estimate total insertion numbers.

## Results and Discussion

### Pogostick main features

*Pogostick* was designed to facilitate cloning of candidate genes to produce either over-expression or down-regulation vectors. For the construction of over-expression vectors, the full-length cDNA of the target gene should be inserted into either the 5′-MCS, the 3′-MCS, or into both sites (Fig. 1B). For the construction of down-regulation vectors, a cDNA fragment of the target gene should be inserted into both MCS sites, in reverse orientation, to form inverted repeats (IR), hairpin RNA structures that can specifically silence gene expression via the mechanism of RNAi. Pogostick can be found at www.addgene.org, a non-profit plasmid repository.

#### The Multiple Cloning Sites

The design of the two multiple cloning sites of *Pogostick* (Fig. 1B), with carefully chosen RE recognition sequences, give the vector its versatility and easiness of use for multiple candidate genes. Two of these sites, *NheI* (in the 5′ MCS) and *SpeI* (in the 3′ MCS), should be sufficient for most cloning strategies, but other sites were also included as back-up. The basic idea is to use one of a suite of four alternative REs, *NheI* (5′-GCTAGC-3′), *SpeI* (5′-ACTAGT-3′), *AvrII* (5′-CCTAGG-3′) and *XbaI* (5′-TCTAGA-3′), all producing the same sticky ends, 5′-CTAG-3′, to digest any candidate gene fragment before cloning it into *Pogostick*. Each gene fragment, in turn, has one of these sites added to its ends via the use of modified PCR primers (see specific example for *DsRed* below). In addition, the RE chosen for cloning each gene fragment into *Pogostick* should not cut the gene at any other site, aside from the artificially extended ends. For down-regulation vectors, the size of the cDNA fragment should be between 500-1000 bp in length, as this triggers stronger silencing than shorter fragments [41] and should preferably be a single complete exon from the target gene, since exons often contain sequences that facilitate the processing of transcripts [41]. Exons that are known to be alternatively spliced should be avoided, since these might contain silencing sequences that repress or restrict splicing [41].

#### The inducible heat-shock promoter

The heat-shock promoter of *Hsp70* of *Drosophila* and its polyA signal, shown to work across different insect species including mosquitoes, moths, sawflies and butterflies [34], [35], [42], [43] were used as the inducible promoter and transcription termination signal in this vector (Fig. 1A). The temporal control of transgene over-expression and down-regulation via a heat-shock is especially important when the genes in question have multiple functions during development, allowing each of these functions to be investigated separately.

In case the *Drosophila* promoter does not work efficiently in a particular species, as demonstrated in *Tribolium castaneum* [44], it can be replaced using the following steps: The new promoter should be amplified with primers PromoterF (ATTACCTCAGGTC + 10 bp of the most 5′ sequence of the new promoter) and PromoterR (TCGCTTAATTAAGT + the reverse complement sequence of the most 3′ 10 bp of the new promoter) from DNA containing the new promoter sequence. The amplified fragment and the *Pogostick* vector should then be digested with *AocI* and *PacI*, and the digested promoter fragment inserted and ligated into the cut vector. The insertion should be confirmed through 5′ sequencing with primer TCGAGCTTAAGaGATCTGTCA (producing 57 bp of old vector + 5′ end of the new insertion) and/or 3′ sequencing with primer TGACAGATCtCTTAAGCTCGA (producing 10 bp of old vector + 3′ end of the new insertion).

#### The 3*×*P3*−*EGFP marker for transgenesis

The 3*×*P3*−*EGFP cassette that mediates EGFP expression in all larval, pupal, and adult eyes of Diptera, Lepidoptera and Coleoptera tested so far [38], [45], [46] and predicted to work across metazoa with eyes, was used as the marker for transgenesis (Fig. 1A).

#### The *white* intron

An intron (the second intron of the *white* gene from *D. melanogaster*) positioned between the two MCSs (Fig. 1B) has multiple functions. First, it provides an anchor for primers when these are used to check the orientation of the inserted transgenes. Second, the intron stabilizes both the expression of the transgene [47] and the plasmid replication in *E. coli* [41]. Thirdly, having a spacer between the IRs is known to strongly enhance RNAi silencing activity in plants [48] and produce strong and uniform RNAi silencing in *Drosophila* [41]. Flanking the intron sequence we placed consensus sequences that code for short intron splicing throughout all organisms (GCTAGCAG at the 5′-end and GTACTAGT at the 3′-end [49]. These splice sites remove the *white* intron after the mRNA is transcribed (Figs. 1B, 3A, 3C).

In the event that this intron needs to be replaced, the new intron should be amplified with primers IntronF (TTAAGCTAGCAG + 10 bp of the most 5′ sequence of the new intron) and IntronR (CCGGACTAGTAC + the reverse complement sequence of the most 3′ 10 bp of the new intron) from donor DNA. The amplified fragment and the *Pogostick* vector should then be digested with *NheI* and *SpeI*, and the digested intron fragment inserted and ligated into the cut vector. The insertion should be confirmed through 5′ sequencing with primer CAAGCGCAGCTGAACAAGCTA (producing 187 bp of old vector + 5′ end of the new intron) and/or 3′ sequencing with primer AGAATGTAGAATGAACCCATGT (producing 368 bp of the old vector + 3′ end of the new intron).

### Construction of DsRed specific vectors

The cloning strategy outlined above, using only *XbaI*, *NheI*, *SpeI or AvrII* REs was successfully applied to construct *DsRed* and *Ubx* over-expression vectors using both of the *Pogostick* MCS (*Pogostick-up-1*, *Pogostick-up-2*) and *DsRed* down-regulation vectors (*Pogostick-down-FR* and *Pogostick-down-RF*). Subsequently, other over-expression and down-regulation vectors were also constructed using the same cloning strategy, suggesting its wide applicability for different genes. Over-expression vectors were made using the 1077 bp full-length *Distal-less* CD (AF404825) and the 1059 bp full-length *engrailed* CD (unpublished) of the butterfly *Bicyclus anynana*. And down-regulation vectors were constructed with CD fragments of *wingless* (459 bp, unpublished), *decapentaplegic* (437 bp, unpublished) and *spalt* (140 bp, unpublished) of *B. anynana*.

### Over-expression assays in Drosophila

**Figure 4:**
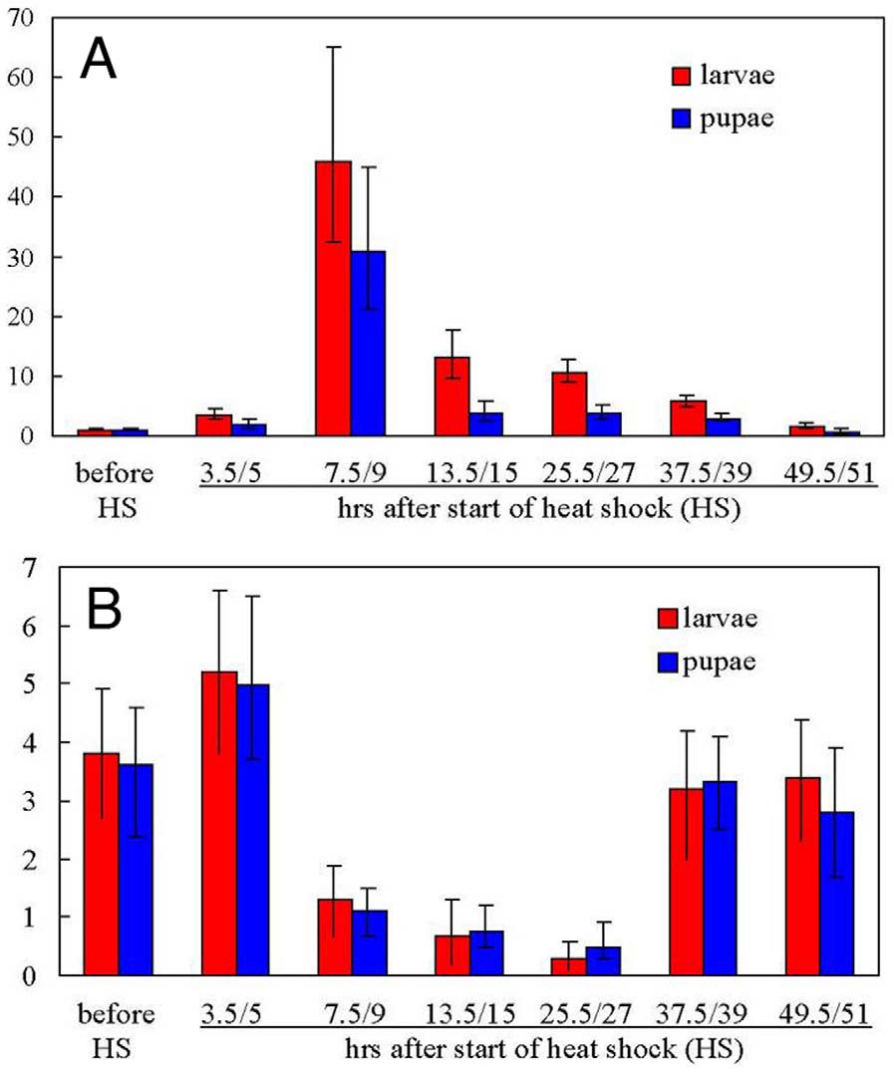
Heat-shocks lead either to *DsRed* mRNA over-expression or down-regulation in *Drosophila* larvae and pupae. Quantitative RT-PCR analysis of *DsRed* mRNA levels in *D. melanogaster* in an over-expression line *Pogostick-up-1* (A) and down-regulation line (B; resulting from a cross between *pBac[3×P3−DsRedaf]* and *Pogostick-down-RF* homozygous parents). Relative quantification in 2-^ΔΔC^_T_ indicate the levels of *DsRed* transcript normalized to the internal standard 18S rRNA. Error bars indicate the range of minimum and maximum levels of four repeats. Larvae were heat-shocked for 1.5 hrs at 39°C, and pupae for 3 hrs at 39°C.

**Figure 5:**
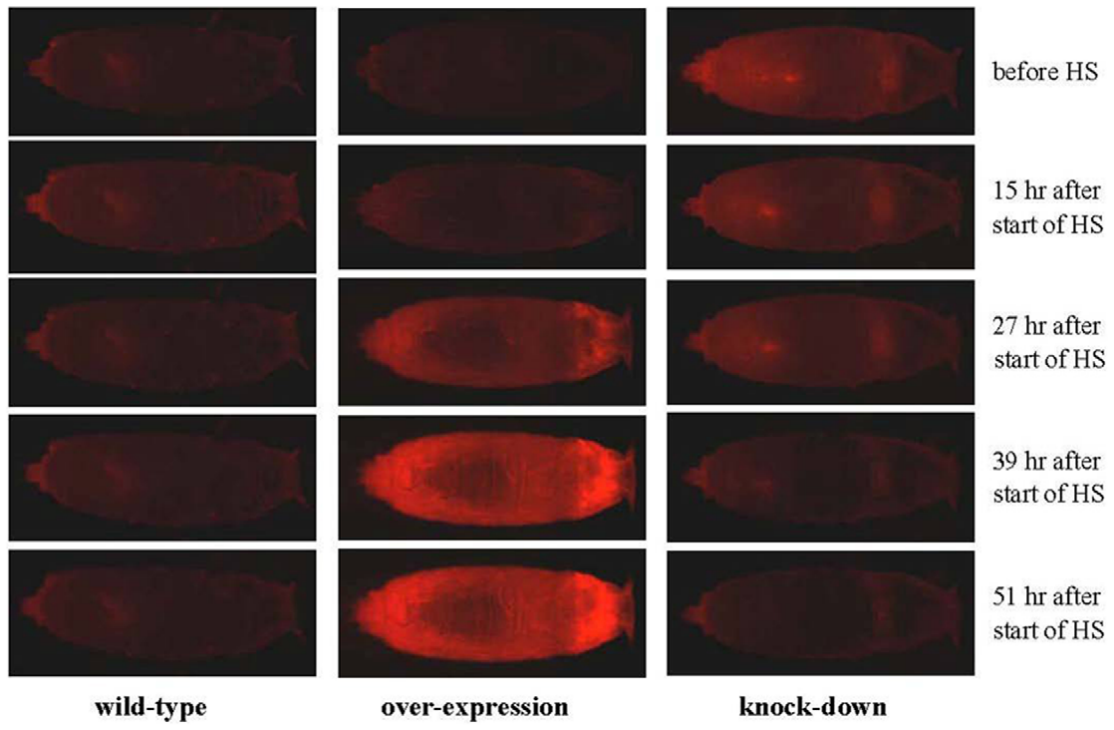
DsRed fluorescence following a heat-shock in *Drosophila* pupae. Heat-shocks lead either to increased (A) or decreased (B) DsRed protein levels. Pupal phenotypes of wild-type, over-expression (*Pogostick-up-1*), and down-regulation (resulting from a cross between *pBac[3×P3−DsRedaf]* and *Pogostick-down-RF* homozygous parents) lines targeting the *DsRed* gene in transgenic *Drosophila*, before and after a heat-shock (HS). Fluorescence in wild-type lines remained constant, whereas DsRed levels visibly increased or decreased 27 hrs and 39 hrs after the beginning of the HS, respectively.

*DsRed* mRNA was similarly over-expressed in both *Drosophila* transformed *Pogostick-up-1* (Fig. 4A) and *Pogostick-up-2* lines (not-shown), induced by 1.5 hrs of heat-shock at 39°C for larvae and 3 hrs for pupae. mRNA levels were at their maximum at the 7.5 hrs (45 fold higher) and 9 hrs (30 fold higher) sampling points after the beginning of the heat-shock for larvae and pupae, respectively (Fig. 4A). Levels declined with time and returned back to pre-heat-shock levels at 49.5 hrs and 51 hrs for larvae and pupae, respectively. Protein levels were visibly elevated at 27 hrs after the beginning of the heat-shock for pupae, as detected by fluorescence microscopy (Fig. 5). Protein levels continued to visibly rise from 27 hrs to 51 hrs, indicating that DsRed protein was not readily degraded (Fig. 5). There appears to be an 18 hrs time lag for DsRed mRNA transcripts to produce a functional fluorescing protein. Larvae showed a similar protein expression pattern to the pupae (not shown). Three hour heat-shocks (applied to pupae) did not appear to produce more extreme mRNA or protein expression levels as compared to 1.5 hrs heat-shocks applied to larvae. There was no change in levels of red fluorescence in either the *Pogostick* control vector lines or in wildtype flies (Fig. 5) with the heat-shock treatments. *Pogostick* control lines constitutively expressed the EGFP marker in the eyes (not shown).

### Down-regulation assays in Drosophila

*DsRed* mRNA levels were significantly reduced 7.5 and 9 hrs after the beginning of the heat-shock for larvae and pupae, respectively (Fig. 4B). mRNA levels continued to gradually decline until 25.5 hrs or 27 hrs for larvae and pupae, but returned to pre-heat-shock levels at 37.5 hrs and 39 hrs for larvae and pupae, respectively. The slight increase in mRNA levels immediately following the heat-shock could represent detection of the hairpin-loop dsRNA structure before the RNAi mechanism takes effect (Fig. 4B). Protein levels declined slightly from 15 to 27 hrs and declined more abruptly from 27 to 39 hrs after the beginning of the heat-shock for pupae, as detected by fluorescent microscopy (Fig. 5). Levels remained low at 51 hrs after the heat-shock (Fig. 5).

There appears to be a delay of around 30 h from the moment low mRNA levels are detected (9 hrs) to visibly reduced protein levels (39 hrs). Larvae showed a similar protein expression pattern to the pupae (not shown). Three and 1.5 hrs heat-shocks produced similar results.

### Correct intron splicing

The correct splicing of the *White* intron was verified by sequencing the mature mRNA transcripts of *DsRed* over-expression and down-regulation *Drosophila* transformed lines. The sequence was confirmed to be that shown in Figs 3B and 3D.

### Flanking sequences to Pogostick insertions

Five independent genomic sequences adjacent to piggyBacL TIR were recovered from *Pogostick, Pogostick-up-2* and *Pogostick-down-RF* lines, using the unique RE site *BstSNI*. All of these sequences contained genomic DNA flanking the TIR, with the signature TTAA sequence at the integration site (Table 1). Corresponding genomic sequences adjacent to piggyBacR and confirmation of TTAA duplications were also obtained using the unique RE site *BbvCI* (Table 1). The detection of piggyBacL adjacent sequences was more effective than the detection of piggyBacR adjacent sequences, as the produced plasmids were relatively smaller. Therefore, the detection of piggyBacL-adjacent genomic sequence with *BstSNI* is recommended.

**Table 1:** *PiggyBac* transposition in *Drosophila* germlines.

Germline	FGSL	FGSR	Chromosome	Gene Name	Insertion Position
*Pogostick*	AGGTTGTCGG**TTAA**	**TTAA** CCTCAGGTCT	X		intergenic
*Pogostick-up-2*	AGCATATTAT**TTAA**	**TTAA** TTGCGTTTAT	2L	CG7261-PA	intron
	CAGACACATT**TTAA**	**TTAA** TGATGCATGC	3R		intergenic
*Pogostick-down-RF*	AATTTATATA**TTAA**	**TTAA** TTTTTATCAT	3R		intergenic
	GTTCATGTAG**TTAA**	**TTAA** CTTGTTTTGT	U		intergenic

The flanking genomic sequences obtained with insertion site TTAA on the *piggyBacL* and *pigyBaR* are shown separately as FGSL and FGSR.

### Producing homozygous lines

In the current manuscript we used *Drosophila* genetic tools (balancer chromossomes) and levels of eye fluorescence in *Bicyclus* to produce homozygous transgenic lines. In the event that levels of eye fluorescence are difficult to distinguish between homozygous and heterozygous individuals, it is still possible to generate a homozygous line using the identified genomic flanking sequence information in multiplex PCR assays [50]. Primer pairs can be designed for the new genomic regions (A and B, pointing towards each other) and a separate primer can be designed for a nearby flanking *Pogostick* sequence (primer C, close to and pointing towards A, or close to and pointing towards B). In heterozygous individuals it should be possible to recover PCR amplicons of AB and AC (or BC), whereas in homozygous individuals it should only be possible to recover amplicons of AC or BC. Wt individuals should have AB amplicons only.

### Over-expression assays in Bicyclus

**Figure 6:**
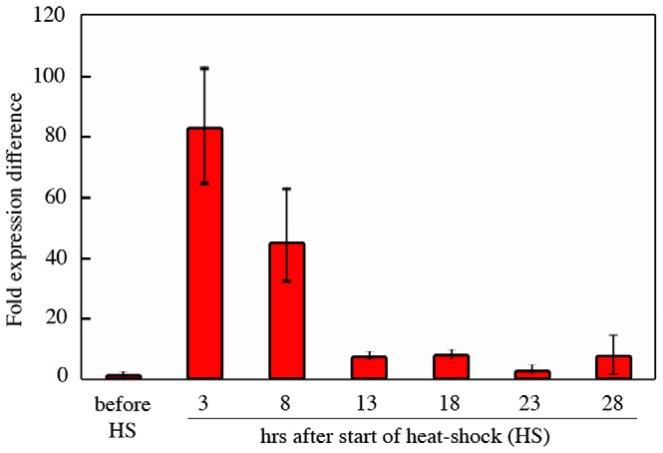
Levels of *Ubx* mRNA in transgenic *Bicyclus anynana* larvae before and after a heat-shock. A single 3 hr heat-shock lead to nearly a 80 fold increase in *Ubx* levels in *Bicyclus* larvae. Note that pre heat-shock levels are near zero because the levels we are measuring correspond to *Junonia Ubx* (the transgene used), rather than *Bicyclus Ubx.*

*Ubx* mRNA levels were at their maximum immediately after the end of the heat-shock (3 hrs) (80 fold higher relative to non-heat-shocked controls) (Fig. 6). Levels declined with time and returned to close to pre-heat-shock levels at 23 hrs after the beginning of the heat-shock. The accelerated production of *Ubx* mRNA in *Bicyclus* relative to *DsRed* mRNA in *Drosophila* may have to do with the higher rearing temperature used for *Bicyclus* (27°C relative to 20°C for *Drosophila*). This may also explain why the degradation of the mRNA for *Bicyclus* also happened at about twice the speed as that observed for *Drosophila*.

In conclusion, the new *piggyBac* vector *Pogostick* has the following traits: 1) it can be used to induce the over-expression and down-regulation of candidate genes in a temporally controlled fashion by means of a heat-shock and; 2) it allows for the easy characterization of the genomic insertion site and copy number. The vector is available to anyone wanting to test it in different organisms. Future improvements to this vector may include addition of insulator elements to the ends of the piggyBac TIRs that may insulate the expression of the vector from position effects [51] and/or the addition of a *attP* site that will later allow the very efficient phiC31 integrase-mediated recombination to modify, replace or stabilize transgenes at the same genomic location [52], [53].
